# Making the Nēnē Matter: Valuing Life in Postwar Conservation

**DOI:** 10.1093/envhis/emaa002

**Published:** 2020-06-09

**Authors:** Duncan Wilson

## Abstract

In 1950, a group of scientists and public figures, based in Hawaii and England, launched a transnational “restoration project” to save the nēnē or Hawaiian goose from extinction. Scrutinizing this project highlights how endangered species were valued as part of a historically contingent process that reflected and linked the interests of different groups. People did not undertake the restoration project simply because they realized the nēnē were endangered, but, instead, they sought to rescue it at the “eleventh hour” in order to legitimize the new conservation organizations that they helped establish after the Second World War. They also engaged with broader political and socioeconomic concerns to justify the restoration project, publicly framing the nēnē as a valuable asset that benefited Hawaii’s tourist economy and push for statehood. Disputes over the reintroduction of geese bred in England highlight how the nēnē were valued in complex and sometimes contradictory ways, with unforeseen consequences for both the restoration project and its animal subjects. This case study ultimately draws our attention to the inherently biopolitical nature of modern conservation, by showing that there is no simple trajectory from endangered life to valued life.

## INTRODUCTION

In a 1945 survey for *Condor*, the zoologist Paul H. Baldwin reported a dramatic reduction in “numbers and range” of the nēnē or Hawaiian goose, the largest native land bird found on the Hawaiian Islands.^1^ This medium-sized goose, with fawn, brown, and black plumage, long black legs, reduced foot webbing, and a black bill, was well known for flocking in large numbers across high altitude and lowland areas on the islands of Hawai‘i and Maui during the nineteenth century. By the 1930s, however, the nēnē had disappeared from Maui, and National Park staff estimated that no more than fifty remained on the island of Hawai‘i. Drawing on testimony from park rangers, ranchers, and “other outdoor observers,” Baldwin claimed that the nēnē had become a “rarity” because its distinctive lifecycle rendered it vulnerable to changes that accompanied the expanding Euro-American presence on the islands from the 1790s onwards.^2^

Nēnē differ from other goose species in that they mate on land and build their nests under bushes. The goslings that hatch after around thirty days of incubation cannot fly for the first eleven to twelve weeks of life, during which period both adults molt their wing feathers and are flightless for six weeks.^3^ Baldwin outlined how many nesting sites were destroyed to make way for farms, sugarcane plantations, resort homes, and military roads, and he detailed how growing numbers of “introduced animals,” such as feral dogs and rats, killed adults and goslings during their long flightless spell.^4^ The most destructive new species was the mongoose, which had been introduced in 1883 to stop rats from damaging sugarcane but which soon began attacking the nēnē. The decline in many bird species was hastened once white settlers began hunting them with shotguns for sport, and the nēnē were particularly easy quarry not only because of their long nesting season and molt but also because they either froze or retreated to an elevated position when approached. Baldwin warned that this combination of “adverse factors” ensured that “the Nene faces an uncertain future.” Unless “means may be found to preserve the species,” he concluded, “there is little prospect it will survive the present development of the island.”^5^

But the nēnē did ultimately survive, thanks to a transnational conservation effort that began in 1950. This so-called “restoration project” involved captive breeding at specialized facilities in Hawaii and England and the introduction of artificially reared nēnē to nature reserves on the islands of Hawai‘i and Maui.^6^ Contemporaneous scientific papers, official reports, newspaper articles, and archived correspondence suggest that the nēnē’s defenders did not undertake or seek to justify this project simply because the geese were endangered. At-risk status alone was not sufficient to spur protection. As Judith Butler has claimed of threatened human populations, “it does not follow that if one apprehends a life as precarious one will resolve to protect that life or secure the conditions for its persistence and flourishing.” Butler instead has argued that precarious life is always viewed through epistemological frames that are “politically saturated” and embody particular beliefs or assumptions, with these “operations of power” ultimately determining whether or not the life in question is worthy of preservation. The value of precarious life only appears, she concludes, under conditions in which its loss is seen to matter.^7^

Although the animal studies scholar Cary Wolfe criticized Butler for restricting her analysis to human lives, Charis Thompson and Thom Van Dooren have examined the ways in which the same logic has shaped efforts to protect endangered species such as the elephant and the Hawaiian crow (*‘alalā*). They argue that “it is not rarity in any objective sense” that has determined conservation priorities but rather the ways in which rarity is filtered through the prism of institutional, political, or social commitments.^8^ A close study of efforts to preserve the nēnē underscores the degree to which this proved to be the case for what would become Hawaii’s state bird.

The ostensible impetus for the nēnē “restoration project” came initially from a new Severn Wildfowl Trust, an English conservation organization established by the painter and broadcaster Peter Scott in 1946 to “arrest the decline in the world’s wildfowl.”^9^ During a period when new conservation organizations increasingly drew attention to the plight of endangered species, Scott believed rescuing the nēnē “at the eleventh hour” would help legitimize and promote work in his Wildfowl Trust and world conservation more generally.^10^

Scott and colleagues in Hawaii sought to gain funding and support for their work by publicly framing the nēnē as a valuable economic, social, and political asset. They lobbied for its adoption as the official bird of the Hawaiian Islands and argued that it was a “unique and native” species that should be preserved “as something of ‘Old Hawaii’ for the education of tourists and local residents.”^11^ These claims dovetailed with, and helped perpetuate the ongoing production of, what Cristina Bacchilega calls “legendary Hawai‘i,” which involved the reimagining and marketing of “native” traditions and natural resources for a booming tourist economy.^12^ But the presentation of the nēnē as a valuable emblem of “Old Hawaii” had unintended consequences that impeded the ambitions of some participants in the restoration project. Scott’s efforts to introduce nēnē bred at the Severn Wildfowl Trust to Hawaii were frustrated after federal authorities considered them a potential source of disease and threat to the now cherished native population. At a time when conservation organizations prioritized saving animals in their natural habitat, emphasizing the nēnē’s “nativeness” threatened to devalue these English-bred geese to little more than museum specimens.

Detailing how these individuals and organizations made the nēnē matter reframes some important themes in the history of conservation. Historians often evoke the concept of nonhuman charisma when they explain why certain animals are prioritized in wildlife conservation. They present ideas about charisma as being tied to the social construction of nature and detail how endangered species were valued as the embodiment of specific norms and virtues, such as the “rugged” bison that were preserved as symbols of the disappearing American frontier during the late nineteenth century, or as emblems of particular regions and nation-states, such as the pandas that became tools for Chinese patriotism and diplomacy in the twentieth century.^13^ These histories show that charisma and value are historically contingent human inventions that intersect with, and are themselves shaped by, social, political, and economic factors. Yet they generally portray charisma and value as prerequisites for conservation, exploring how scientists and politicians sought to rescue animals that were already valued for specific reasons. Studying the nēnē “restoration project” demonstrates, however, that value can be manufactured for a species after people realize it is endangered in order to validate their work, and it shows that some endangered animals can also lose value thanks to the ongoing “ideological mediation” that links conservation to broader ideas surrounding the “native” or “tradition.”^14^ While we need to be mindful of the ways in which these values are historically, socially, and locally rooted, accounts such as this can nevertheless contribute to present-day concerns about conservation priorities and species loss by reminding us that there is no simple trajectory from endangered life to valued life.

## THE NĒNĒ AND POSTWAR CONSERVATION

Peter Markham Scott was born at Buckingham Palace Road, London, in September 1909, the only child of the explorer Robert Falcon Scott and the sculptor Kathleen Scott (née Bruce). Shortly before he died during an expedition to the South Pole in March 1912, Scott’s father wrote home and urged his mother to “make the boy interested in natural history.” With a place in elite society guaranteed thanks to a sizeable memorial fund and his mother’s success as a sculptor, Scott had more opportunities than most to explore the natural world. He was made a Life Fellow of the Zoological Society of London as a christening present and received private tutorials from renowned biologists, who he recalled “were prepared to give time to me because of the passage in my father’s letter.” By the time went to preparatory school in Winchester at the age of ten, Scott was “deeply committed to Natural History.”^15^

Scott’s fascination with geese started when he attended boarding school in Cambridgeshire and began sketching the grey geese found on local floodwaters. After arriving at Trinity College, Cambridge, to read natural sciences in 1927, Scott became a “fanatical wildfowler,” who divided his time between painting and shooting geese. Like others who viewed wildfowling as a restorative pursuit, Scott had no trouble reconciling his passion for hunting and his “love of living birds.” Geese were “man’s traditional quarry,” he argued, “and it was part of man’s instinct to hunt; it was part of the bird’s instinct to be hunted.”^16^

After training in Munich and the Royal Academy of Arts in London, Scott began his career painting wildfowl in 1933 and set up home in a converted lighthouse at the mouth of the River Nene in Norfolk. Scott’s enthusiasm for hunting was gradually replaced by a desire to keep birds alive and study them. He became proficient in catching wild birds and traveled across the United States and Europe to acquire rare species for the growing collection he kept in an enclosure on the marshes surrounding his lighthouse. Establishing connections with private collectors and the directors of wildlife reserves, he increasingly combined a scientific and artistic persona: detailing the habits and management of captive and wild birds as he painted them.

In 1938, Scott learned that the wealthy rancher Herbert Shipman possessed the world’s only captive nēnē population on his property in Hawai‘i. The two corresponded, and Shipman agreed to provide Scott with a breeding pair if he collected them personally.^17^ Before Scott could travel to Hawai‘i, however, Britain declared war on Germany, and he was called up to the Royal Navy, temporarily distracting him from the project. Indeed, it would be almost a decade before Scott returned his attention to importing nēnē, and, when he did so, he sought to acquire them for a different location and under the guise of a conservation organization rather than as an individual. Shortly after the war ended, Scott visited the River Severn in Slimbridge, Gloucestershire, searching for a rare lesser white-fronted goose that had been spotted by local birdwatchers. After sighting the goose among a large flock on the estuary saltmarshes, he decided to make Slimbridge the location of a new establishment dedicated to the scientific study, public display, and conservation of wildfowl.^18^

This Severn Wildfowl Trust was formally established in November 1946, with Scott as director and an advisory council that included the ornithologist and civil servant Max Nicholson. While the grounds were landscaped, and Scott built up a collection that included sixty-seven different species, council members set out the trust’s aims during their first annual general meeting. They agreed that its work was to be “in part educational and in part scientific,” allowing paying visitors to view wildfowl and hiring staff to undertake research on the tame collection and the flocks that visited the grounds each year.^19^ Both aspects, in turn, fed into the trust’s overall goal of helping to “arrest the decline in the world’s wildfowl.” The first booklet that the trust produced to encourage public subscriptions, in 1948, made clear that conservation was its overriding aim. “In nearly all parts of the world,” it argued, “wildfowl—ducks and geese—are declining in numbers. … If they are to survive, certain steps will need to be taken, and in order to make sure these steps are the most helpful ones, much research is still needed into the birds [*sic*] habits, life histories and migration routes.”^20^

The formation and aims of the Severn Wildfowl Trust reflected and contributed to broader trends. After 1945, elite figures in international organizations such as the United Nations Educational, Scientific and Cultural Organization (UNESCO) claimed that the wasteful exploitation of natural resources had been a “precipitating factor in the previous war” and argued that the rational management of these resources was vital to maintaining international stability in the future.^21^ The biologist Julian Huxley used his position as director general of UNESCO to establish a new International Union for the Preservation of Nature (IUPN) in 1948, which subjected the natural world to unprecedented scrutiny and governance. The IUPN differed from the majority of previous organizations in its emphasis on the need for cutting-edge science and in the way it reframed conservation as a global problem that required international collaboration.^22^

While the IUPN sought to preserve “the entire world biotic community,” it concentrated largely on species known to be at risk of extinction. The major outcome of its first conference in 1949, for instance, were two lists of thirteen birds and fifteen mammals “in need of emergency action if they are to be saved.”^23^ These “emergency” lists were by no means exhaustive and consisted of species that had been extensively surveyed and whose numbers were well known, largely thanks to their status as game animals. They also served a dual purpose: focusing international attention on the species in question and on the IUPN, whose General Assembly claimed it would attract support and funding through demonstrating “concrete accomplishments” in saving these “urgent cases.” ^24^ With this in mind, it is telling that experts who compiled these emergency lists only included species they believed to have a fighting chance of survival and omitted those “whose situation seemed hopeless.”^25^ If saving endangered species was a means of securing attention and support for new conservation groups, there was little point in championing lost causes.

The nēnē appeared on the IUPN’s bird list in 1949, indicating that not everyone shared Baldwin’s pessimism about its chances of survival. Figures at the Severn Wildfowl Trust also believed the nēnē could be saved and argued that rescuing it “fell within the scope of the objects for which the Trust was formed.” Like the IUPN, they felt that “making a practical contribution to the preservation of a vanishing species” would demonstrate the importance of their work.^26^ Scott reignited his interest in the nēnē soon after the Severn Wildfowl Trust was established in 1946, asking the territorial government in Hawaii what steps they were taking to save the fifty or so remaining geese. Although this query went unanswered, Scott was presented with a chance to intervene in 1948 when he corresponded with the American biologists Charles and Elizabeth Schwartz, who had recently been invited to Hawaii to advise on the management of game birds. The Schwartzes were unsure about the job, but Scott urged them to accept in order to draw further attention to the nēnē’s plight.^27^

**Figure 1.**
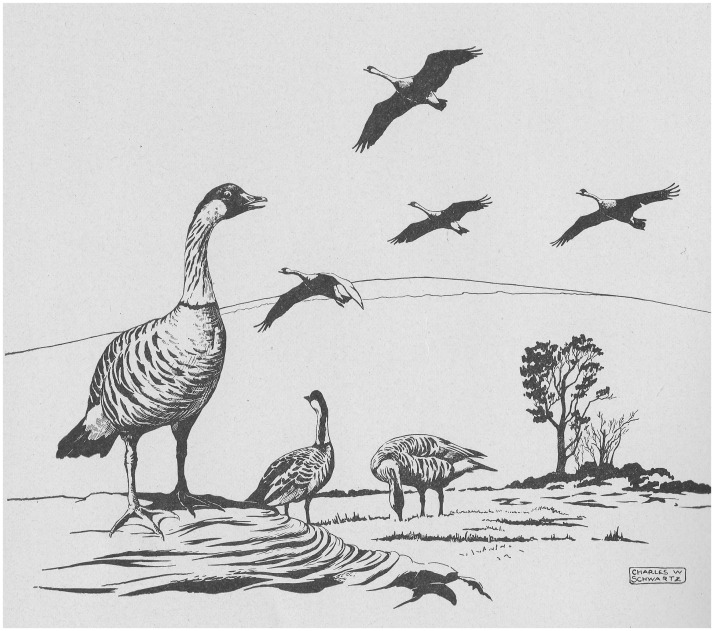
A flock of nēnē on lava flow, drawn by Charles Schwartz. Credit: Charles W. Schwartz and Elizabeth Reeder Schwartz, *A Reconnaissance of the Game Birds of Hawaii* (Territory of Hawaii: Board of Commissioners of Agriculture and Forestry, 1949).

Published in 1949, the Schwartzes’ report detailed how “high on the volcanic slopes of Mauna Loa and Mt. Hualalai, a remnant flock of probably the world’s rarest bird ekes out its precarious existence” (figure 1). They claimed that the nēnē were still threatened by hunting, despite a ban, as well as by “mongooses, rats, cats, dogs, pigs, grazing livestock, the changing vegetative pattern, and land use practices.” Predicting it was “the next Hawaiian, if not world, species facing imminent extinction,” the Schwartzes argued that permitting “this tragedy to occur without exerting more effort than has been done to date is unpardonable.” They also acknowledged that traditional conservation measures such as preventing hunting and controlling predators would not “restore the species” in light of its precarious numbers and extensive range. The “only practical means for its restoration,” they concluded, involved securing “breeding stock from captive birds and propagating this species as intensively as possible.”^28^

Territorial authorities responded to the Schwartzes’ proposals by allocating six thousand dollars for a breeding project run by the ornithologist J. Donald Smith at the Forestry and Fish and Game Camp in Pohakuloa, a plateau stretching between the lower slopes of Mauna Loa and Mauna Kea in Hawai‘i. Herbert Shipman loaned Smith two pairs of nēnē, which were housed in large pens reinforced with sheet iron to stop predators and fed a diet of grains, turkey pellets, and common sow thistle. The small captive flock was supplemented early in 1950 by a wild-caught female and a male sent from the Honolulu Zoo as its mate. Although Smith claimed that “the major objective of this propagation venture is to produce 50 geese a year to be released into the wild,” the initial results proved disappointing.^29^ One of Shipman’s females died before nesting, and his other pair produced only two goslings from four eggs. The third pair produced no fertile eggs, and the male, which turned out to be sterile, was returned to the zoo.

Keen to “step up the production rate,” Smith sought advice from former colleagues at the Delta Wildfowl Foundation, an organization dedicated to waterfowl research based on Lake Manitoba in Canada, who suggested he consult breeding experts at the Severn Wildfowl Trust.^30^ After being invited to help the restoration project, the trust sent their curator, John Yealland, to Hawai‘i in 1950. Yealland advised Smith to implement the trust’s standard rearing protocol, which sought to increase hatching rates by removing the first clutch of eggs from geese and placing them under surrogates. Once their eggs were removed, a high proportion of breeding pairs mated again, and Yealland recommended that Smith leave the nēnē to incubate this second clutch themselves.

**Figure 2.**
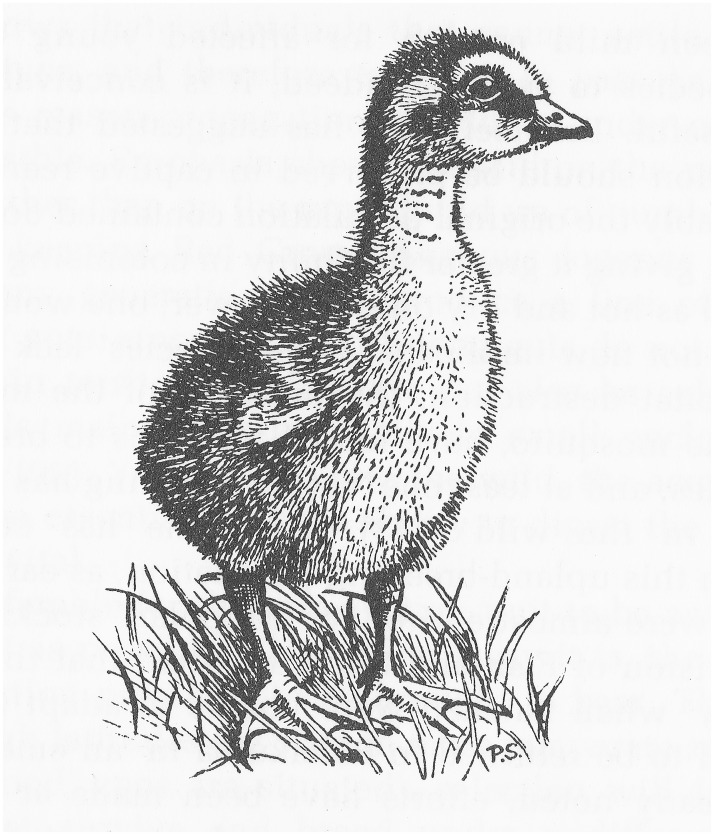
One of the first nēnē goslings hatched at Slimbridge, drawn by Peter Scott in 1952. Credit: Peter Scott, “The Breeding of the Ne-Ne or Hawaiian Goose.” Courtesy of the Wildfowl and Wetlands Trust and the estate of Sir Peter Scott.

Before Yealland left Hawai‘i, Shipman gave him a pair of nēnē to take back to Slimbridge. Mindful that a tidal wave had killed thirty of Shipman’s flock in 1949, Hawaiian authorities believed sending nēnē abroad provided “insurance against the entire loss of breeding stock due to complete destruction of geese at Pohakuloa,” and they also hoped the trust’s aptitude for breeding waterfowl would generate new findings that would help them attain their own “production objective.”^31^ Work at Slimbridge got off to an inauspicious start, however, when both nēnē laid infertile eggs, and it became clear that Shipman had donated two females. After receiving an urgent telegram asking for a male bird, Smith sent the Severn Wildfowl Trust one of the two males Shipman had loaned the Pohakuloa project. In February 1952, both females mated with the new arrival and laid fertile eggs. Once the trust staff removed the eggs and incubated them under bantam hens, the females laid again and were left to incubate the second clutch. At the end of the first full breeding season at Slimbridge, these efforts led to the successful hatching of nine goslings from nineteen eggs (figure 2).^32^

In the Severn Wildfowl Trust’s fifth annual report, Scott claimed that the twelve nēnē housed at Slimbridge “probably represent about twenty per cent of the world population.” Given that council members viewed conservation as the trust’s main objective, he unsurprisingly considered “the successful breeding of the ne-ne” to be its “most important achievement.”^33^ The nēnē’s value was evident in, and reinforced by, the care and attention it received.Trust staff gave females hormones to stimulate egg production, meticulously scrutinized the health of adults and goslings alike, recorded signs of illness, and provided treatment when needed. When one of the females that Shipman provided suffered a prolapse during laying, vets from Bristol University came out “within the hour and carried out a skilful repair,” before taking the goose “for further treatment to the veterinary department where she recovered rapidly.”^34^

But not all species drawn into conservation projects received equal care or attention. The hens and Muscovy ducks used as surrogates in Pohakuloa and Slimbridge functioned as what Van Dooren calls “sacrificial populations” and were valued solely for the role they played in propagating the nēnē.^35^ Correspondence between staff in both locations made clear that these species were monitored primarily to assess their “brooding ability.” Individuals graded “good or excellent” were repeatedly used, while those “responsible for deserting eggs, smothering young, or otherwise lowering productivity were destroyed.”^36^ These practices embodied what Van Dooren identifies as the “violent care” of conservation and highlight the degree to which other species had to be subordinated and sometimes “made killable” in order for the nēnē to survive.^37^

Crucially, the early success in breeding nēnē at Slimbridge gave Scott the chance to publicly endorse both the Severn Wildfowl Trust and a more interventionist approach to conservation. In a 1952 article for *The Times*, he estimated that no more than “32 adult Hawaiian geese are known to exist at the present moment” and argued that “drastic steps were needed” if it was to survive. Indeed, Scott contended, traditional conservation measures, such as banning hunting, establishing nature reserves, and hoping populations recovered naturally, were unlikely to rescue “a threatened species at the eleventh hour.” He claimed that “some form of artificial propagation was clearly desirable,” and he detailed how workers in Pohakuloa and Slimbridge removed eggs from captive geese, incubated them under surrogates, and helped rear the “delightfully tame” nēnē goslings. “Such methods,” he concluded, “might have saved the Passenger Pigeon, the Labrador Duck—even the Dodo. The Hawaiian Goose is not the only bird to which they should be applied today.”^38^

Like prominent figures at the IUPN, Scott and Smith believed that conservation necessitated the “intelligent management” of an endangered species in the wild and, if necessary, in captivity: monitoring its numbers, health, and illness, providing care when needed, and manipulating reproduction to realize its “productive potential.”^39^ By the early 1950s, the nēnē mattered to them because it appeared to vindicate this new and ambitious view of wildlife conservation.

## “SOMETHING OF ‘OLD HAWAII’”

In order to attract funding and support for their restoration project, Scott, Smith, and others also had to outline why the nēnē were worth saving. Their efforts to assert its value reveal how authorities in this period justified conservation in broadly anthropocentric terms, with endangered animals framed as good for people, rather than as valuable in and of themselves, and in ways that reinforced the cultural, social, and economic concerns of particular groups.^40^ This was the case in Britain, where the establishment of the Severn Wildfowl Trust coincided with the start of Scott’s work for the British Broadcasting Corporation (BBC) in 1947. From the early 1950s Scott used his position as the host of the BBC’s natural history programs to endorse the conservation of endangered species. On radio, television, and in public talks, he argued that conservation worked for “the long term benefit of mankind” by saving animals that were vital economic, aesthetic, and scientific resources.^41^ Portraying conservation in this light helped Scott justify ventures like the Severn Wildfowl Trust as a “sort of community chest for saving the world’s wildlife and wild places—things of *value* to mankind.”^42^

Unsurprisingly for an artist, Scott dwelt on the aesthetic reasons for saving endangered species and regularly claimed that nature gave “pleasure, inspiration and a sense of wonder to mankind.”^43^ He argued that contact with, and appreciation of, wild animals provided an antidote to the pressures of modern civilization, and, employing the gendered language of the day, he insisted that when “man cuts himself off from nature something vital inside him shrivels up and dies.” A regular theme in his broadcasts and talks was that wild animals enriched people’s lives, and he consistently maintained that “we of this generation have a responsibility to hand them on to the next.”^44^ The nēnē offered a case in point. In *The Times*, Scott portrayed it as a “beautiful and interesting goose” thanks to its unique lifestyle and “strikingly handsome appearance.” The extinction of “this rare and lovely bird” represented a considerable loss to future generations and saving it was, therefore, “infinitely worth the effort.”^45^

People who promoted the restoration project in Hawai‘i aligned the nēnē’s value with more tangible socioeconomic and political interests. Donald Smith, for instance, argued that efforts to save the nēnē were a “worthy cause” because they allowed the preservation “of something of ‘Old Hawaii’ for the education of tourists and local residents alike.”^46^ This claim drew on and furthered the framing of nature and tradition that portrayed “legendary Hawai‘i” as a pristine and unchanging “slice of the past” for the benefit of a growing tourist economy.^47^ By dwelling on its status as part of “Old Hawaii,” Smith positioned the nēnē alongside other native inhabitants, such as grass-skirted hula dancers, that tourist companies claimed gave tourists an authentic counter to, and refuge from, the modern world.

These ideas about natives and “Hawaiian-ness” functioned as “invented traditions” that were deployed to further the interests of certain professional or social groups.^48^ Claiming that the nēnē were potentially of value to the tourist industry, which politicians viewed as central to Hawaii’s economic fortunes, allowed Smith to portray them as an important cultural and economic asset that warranted conservation. Newspapers agreed. The *Honolulu Advertiser*, for instance, contended that while the “struggle to save the nene is a difficult one, the fact that this grand bird should be saved as a living example of Hawaiian tradition needs to be explained to every schoolchild and teacher, to every cowboy and rancher, to every hunter and sportsman.”^49^

Leveraging the nēnē’s association with “old Hawaii,” conservationists sought to raise its profile further by proposing that it be made the official bird of the islands. In 1957, Paul Breese, director of Hawaii’s Board of Public Parks and Recreation, wrote to Scott suggesting this idea. “The more we think of making the nene the official bird,” he began, “the better the idea seems for several reasons. From the standpoint of popular education, in making the bird more appreciated and valued, and just to generally focus attention on it.”^50^ Breese believed adopting the nēnē as Hawaii’s official bird would help raise funds for work at Pohakuloa and ensure that hunters restrained themselves from shooting wild geese. The brief resolution, which Breese submitted to the Hawaiian Conservation Council in February 1957, claimed that the nēnē was a logical choice as official bird since it was a “unique, native” goose that had “already received international recognition and identification with Hawaii.” It also argued that the move would benefit the nēnē by bringing “valuable publicity and recognition to this bird species,” thereby increasing its chances of “survival and restoration in the wild.”^51^

Supporters of the resolution also engaged with broader debates about Hawaii’s relationship to the United States. Since their formal annexation in 1898, the Hawaiian Islands had been run by a government administered from the United States and existed as a non-self-governing territory. While white settlers had campaigned for statehood during the early twentieth century to secure profitable tariffs for sugar exports, the years following the Second World War saw a more concerted push from those who sought access to American bank loans in order to capitalize on the burgeoning tourist industry.^52^ Growing numbers of politicians in the United States also favored statehood by the 1950s, believing that incorporating a multiracial territory such as Hawaii would improve relations with Asia and allow them to disavow accusations of racism.^53^ Amidst these calls for statehood, Breese and others noted that the mainland states and Alaska, soon to become the forty-ninth state, all had official birds. Indeed, their resolution began by noting that Hawaii was alone in lacking an official bird, and the *Honolulu Star-Bulletin*, which regularly published articles promoting statehood, claimed that adopting the nēnē would ensure the territory “got in step with the 48 states and Alaska, all of which have had official birds for years.”^54^

After the Conservation Council approved the resolution, the Territorial Senate adopted the nēnē as “the bird emblematic of the territory of Hawaii” in May 1957. The following year, Hawaii’s delegate to the United States Congress, John A. Burns, seized on the nēnē’s new status and introduced a bill seeking federal funds for work at Pohakuloa. Drawing on advice from the zoologist William Elder, who had worked with the restoration project in 1956, Burns told Congress that additional funding was “necessary to save this species and restore it to its habitat.” He outlined how federal funds would pay for the recruitment of new staff, the protection of a major nesting site that Elder had discovered in 1956, the building of a specialized facility where geese could be monitored before release, and the strengthening of “the public relations program leading to greater awareness of the need for protection of the Nene, the official bird of Hawaii.”^55^

**Figure 3.**
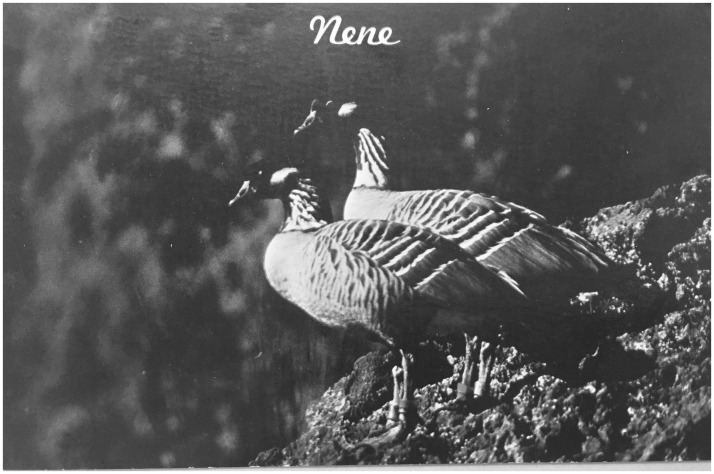
One of several souvenir nēnē postcards sold from 1958 onwards. The colored banding on the legs indicates these were captive bred geese released on either the island of Hawai‘i or Maui. Credit: Syndics of Cambridge University Library.

The bill received nationwide interest, with newspapers aligning the nēnē with debates about statehood. The *Washington Post*, for instance, warned that the nēnē were doomed to “oblivion unless Uncle Sam does something about it” and claimed that Hawaiians “hope still to have some around if and when the territory becomes a state.”^56^ After newspapers and conservation groups endorsed Burns’s proposals, Congress agreed to provide fifteen thousand dollars per year from the budget of the US Fish and Wildlife Service.^57^ This money paid for the recruitment of the biologist David Woodside as project leader and a former poultry superintendent, Ah Fat Lee, as a dedicated nēnē breeder. It also financed the production of souvenir postcards, which Breese predicted would be in “great demand” thanks to the nēnē’s official association with Hawaii (figure 3).^58^

However effective they may have been in securing funding and advancing conservation efforts, these attempts to raise the nēnē’s profile also illustrate how the reimagining of “old Hawaii” excluded the narratives and worldview of those Native people who were simultaneously portrayed, by others, as representatives of Hawaiian tradition. No account that framed the nēnē as a living symbol of old Hawaii detailed how the Native Hawaiians (Kānaka Maoli) had long valued them as *ōiwi* (native of the islands) or how they considered them among the group of significant ancestral animals known as *‘aumakua*, thanks to the use of their plumage in royal standards.^59^ Even though it had been translated into English, none mentioned that nēnē were featured in the *Kumulipo*, an eighteenth-century genealogical chant that documents the creation of the islands along with their indigenous animals. (The nēnē appear in the third chant, which details the birth of fifty-two flying creatures, and are valued as guardians of the sea-dwelling hehe bird.)^60^ Though not necessarily intentional, the absence of Native perspectives here demonstrates that in Hawaii during the 1950s, as elsewhere, not everyone got to publicly define the terms on which endangered species mattered.

## NĒNĒ AND THEIR “NATURAL HABITAT”

The provision of long-term funding, the increase in publicity, and the continued success in rearing goslings at Pohakuloa and Slimbridge fostered optimism about the nēnē’s chances of survival. Shortly after President Dwight Eisenhower signed the Hawaii Admission Act into law in March 1959, making the nēnē the official bird of the fiftieth US state, Scott told Breese that “it really does look as though we will be able to save it now.”^61^ At the end of 1959, with forty-eight nēnē held in Pohakuloa and an estimated world population of 205, leading figures in the restoration project began planning the release of captive-bred geese for what Scott described as “resettlement in the wild state.”^62^

The Hawaiian Division of Fish and Game had already struck an agreement with the landowners for an eight thousand-acre “nēnē sanctuary” on the slopes of Mauna Loa, adjacent to the nesting site that Elder had identified. After Scott reiterated: “there aren’t enough Ne-ne to warrant clumsy experiments with releasing Pohakuloa-raised birds,” Woodside and Breese agreed to populate the new sanctuary using a “gradual settlement technique.”^63^ Handlers placed twenty captive-bred nēnē into a large open-topped pen in March 1960, temporarily clipping their wings to ensure they were confined while they acclimatized and learned to forage for food. Each nēnē was fitted with a colored band for identification, and, as they regained feathers, handlers watched them gradually leave and mix with wild birds before returning to eat and rest in the pen. By late 1960, all twenty nēnē had left the pen for good and established themselves within the sanctuary; some formed mating pairs with geese from Pohakuloa, and others paired with wild geese. This initial release was considered so successful that the Division of Fish and Game quickly secured twelve thousand acres for a second nēnē sanctuary at the base of Mount Hualalai, releasing the first twenty geese there in May 1961.^64^

If the nēnē’s status as an iconic and valued animal was manufactured, so too was the “wild state” into which they were released. As the assistant director of the US Fish and Wildlife Service acknowledged in 1958, “Hawaii is more or less overrun with the mongoose and with feral dogs, cats, pigs and other animals so that any ground-nesting bird is at a very great disadvantage.”^65^ Breese and Woodside decided to safeguard captive bred and wild nēnē by laying poisoned meat to control the numbers of mongoose, wild dogs, and other predators. This policy underscored the degree to which saving the nēnē entailed subordinating animals considered to be less valuable. It also highlighted how ideas about what constituted intelligent management in conservation were not confined to the bodies of endangered species or surrogates but also extended to, and helped reshape, their ostensibly natural habitat.

Thanks in large part to the growth of “invasion biology” from the 1950s onward, much of this management involved removing species designated as “alien” from the environment in which endangered animals either lived or were to be reintroduced, returning it to what the zoo director and writer Gerald Durrell called “something like its former glory.”^66^ Yet ideas about “natives” and “aliens” were not clear-cut and could be reimagined in ways that affected an animal’s value. Disagreement over the fate of nēnē bred at Slimbridge exemplifies the highly contingent way these categories were evaluated.

Like Huxley and other leading figures in conservation, Scott argued that captive breeding should function only as a means of propagating animals that would be eventually used to reinforce wild populations. Until it became clear that some species could not survive in the wild, he believed that breeding endangered animals to keep in captivity was anathema to conservation. This outlook was evident in June 1958, when Scott wrote to Paul Breese stating that there were now enough nēnē at Slimbridge to “start sending birds back to Hawaii for liberation.”^67^ After consulting colleagues in Pohakuloa, however, Breese rejected the offer and replied: “It is our feeling that in view of the strong recommendation by our Veterinarian Pathologist that since both the Nene in the wild and the ones in the breeding project are almost entirely isolated from any other waterfowl, from the possibility of disease standpoint it would be unwise to bring Nene into Hawaii from Europe.”^68^

Breese’s stance refigured the Slimbridge nēnē as “aliens” who harbored a potential threat to the Hawaiian “natives.” In doing so, it undermined the Severn Wildfowl Trust’s hopes of “making a practical contribution” to wildlife conservation. As Geoffrey Matthews, the trust’s director of research, noted in a memo to Scott, “it means that all our efforts have fall [*sic*] into the ‘zoo’ class and we can longer claim to be taking part in a unique conservation programme aimed at re-establishing a vanishing species in the wild.”^69^ In a letter to Breese expressing frustration at the “misguided” decision, Scott reiterated: “Our agreed function was to build an adequate stock in captivity over here, and then to complement the Pohakuloa project in producing young to feed back into the wild—a bold international project of conservation. … We didn’t take Shipman’s birds just to maintain a few living museum specimens.”^70^

The dispute was resolved later in 1958 when Elder suggested using the Slimbridge nēnē to establish a separate flock on Maui. Scott “rather liked the suggestion” and agreed that reintroducing nēnē to Maui provided “further insurance against a catastrophe such as lava flow from Mauna Loa, which would wipe out nearly all the birds if it occurred during the flightless season.”^71^ Once state authorities identified a remote sanctuary location on the edge of the Haleakala National Park, Scott and Breese began planning the first shipment of thirty juvenile nēnē from Slimbridge to Maui.

Nēnē were released on Maui according to the protocol employed in Hawai‘i. Each had its wings clipped, was placed in a release pen, and gradually explored the surrounding area when their feathers grew back, while project staff baited the perimeter of the release site with poisoned meat to kill predators. Although wild dogs killed two females, the Maui release was considered a success, and Scott agreed to provide nēnē from Slimbridge each year. While Hawaiian newspapers presented these “English nene” as “immigrants” returning “to the land of their forefathers,” they did not use their non-native status to raise concerns about the Maui scheme.^72^ Instead, with successive shipments settling in, they began to argue by the end of the 1960s that the “restoration project has been so successful this species has been saved from extinction.”^73^

This became a recurring theme in reports during the 1970s. In 1973, the *Honolulu Advertiser* claimed that the renamed International Union for the Conservation of Nature (IUCN) was preparing to formally downgrade the nēnē from an endangered to a rare species, and it celebrated Scott as “the man who saved Hawaii’s nene goose.”^74^ In 1976, the *National Parks and Conservation Magazine* predicted the nēnē was on its way to becoming a self-sustaining species and detailed plans to establish another flock at the Volcanoes National Park in Hawai‘i.^75^ By the mid-1970s, the Severn Wildfowl Trust had sent over two hundred nēnē to Maui, and, after enquiries from Scott, federal authorities decided that they had enough for restocking needs and gave him permission to start selling goslings to private collectors and zoos.^76^

The nēnē now acquired clear monetary value for the Severn Wildfowl Trust, which had sold 130 breeding pairs by 1980. But its primary value, in England, Hawaii, and elsewhere, remained its status as a symbol of what journalists called “twentieth century man’s emerging concern for the other living creatures that share this earth.”^77^ If these geese were a form of capital, as Scott claimed, then it was often more cultural than financial: helping to raise the status and reputation of those organizations “without whose interest and actions there would be no Nenes left today.”^78^

## CONCLUSIONS

The history of the nēnē restoration project sheds light on some of the major ideas and practices that constitute modern conservation, not least what Matthew Chrulew has called the “prevalence of species thinking.”^79^ In the restoration project, species functioned as “the foundational taxon,” to borrow a term from Harriet Ritvo.^80^ Efforts centered on saving populations that produced fertile offspring, were often restricted to specific habitats, and shared characteristics that differentiated them from other members of a genus. Peter Scott justified the restoration project by dwelling on the nēnē’s distinctiveness, for example, and warned that their extinction represented an evolutionary cul-de-sac that would disrupt “the intricate and delicate web of life” on Hawai‘i.^81^ This worldview meant that endangered species such as the nēnē were afforded a higher priority than that of a subspecies, even when numbers in the latter category were perilously low. The Severn Wildfowl Trust’s efforts to breed the Laysan teal in this period, for instance, received comparatively little attention, even though it was also endemic to the Hawaiian Islands and had a population of no more than thirty-three in 1950, largely because it was considered a “sort of ‘degenerate’ mallard.”^82^ Staff on the restoration project also acknowledged that their efforts to save the nēnē had transformed the species, thanks to intensively breeding from one or two pairs with “reduced genetic variability,” and once the population began to grow significantly in the 1960s they sought to improve it by “eliminating” aggressive individuals and those known to suffer from a genetic condition that impeded the growth of healthy down.^83^ While species was the primary object of concern in conservation, then, it was by no means a fixed category. Indeed, the nēnē were refashioned no less than rescued by the restoration project.

Scrutinizing the restoration project also draws attention to the inherently biopolitical nature of modern conservation, where biopolitics, as Jamie Lorimer has defined it, “describes a modern form of governance that seeks to secure the future of a valued life (both human and nonhuman) at the scale of the population.”^84^ The effort to save the nēnē enriches our understanding of this biopolitical mindset by underscoring the degree to which endangered species are not valued inherently. Designating the nēnē as “endangered” was not enough in and of itself to mobilize the personnel, finances, and political will necessary to “make it live” in biopolitical terms. It had to be shown to matter, and the ways in which its value was asserted reflected and helped consolidate the professional, economic, and political interests of specific groups. The exclusion of Native Hawaiian perspectives in the 1950s and 1960s reaffirms that these values and interests belonged to elite groups that were largely white, male, and Western, leaving little room for the “everyday environmentalism” that emanated from communities without formal connection to professional conservation.^85^

Highlighting how endangered animals are valued in ways that reflect historically contingent beliefs and power relations cautions us against presuming that there can be easy answers to ongoing concerns about widespread species loss today. As Julia Adeney Thomas has noted, different ways of knowing and valuing the world mean “it is impossible to treat ‘endangerment’ as a simple scientific fact.”^86^ There are no objective solutions to the dilemma of which precarious lives warrant protection, and Thomas rightly claims that historians should point scientists and policy-makers toward accounts that demonstrate how lives are valued in complex and often indeterminate ways. Understanding how the values we attach to endangered species are fashioned and negotiated—by whom and for whom—can help us appreciate why some lives continue to take precedence over others in what Van Dooren calls the “edge of extinction.”^87^

## Footnotes

1 Paul H. Baldwin, “The Hawaiian Goose, Its Distribution and Reduction in Numbers,” *Condor* 47 (1945): 27. I use “Hawaii” throughout to refer to the island archipelago and “Hawai‘i” when referring to the single island by that name. I also employ diacritics as they appear in Hawaiian orthography but retain alternative usages as they appear in sources that use other conventions (for example, “Ne-ne” or “nene”).

2 Ibid.

3 Janet Kear and A. J. Berger, *The Hawaiian Goose: An Experiment in Conservation* (Calton: T & A. D. Poyser, 1980), 48–57.

4 Baldwin, “Hawaiian Goose,” 34.

5 Ibid., 35.

6 Ronald L. Walker, “Nene Restoration Project Report,” *Elepaio, Journal of the Hawaiian Audubon Society* 31 (1970): 1–7.

7 Judith Butler, *Frames of War: When Is Life Grievable* (London: Verso, 2011), 2, 14.

8 Thom Van Dooren, “A Day with Crows: Rarity, Nativity and the Violent-Care of Conservation,” *Animal Studies Journal* 4 (2015): 13; Charis Thompson, “When Elephants Stand in for Competing Philosophies of Nature: Amboseli National Park, Kenya,” in *Complexities: Social Studies of Knowledge Practices*, ed. John Law and Anne-Marie Mol (Durham: Duke University Press, 2002), 166–90. For criticism of Butler’s anthropocentric bias, see Cary Wolfe, *Before the Law: Humans and Animals in a Biopolitical Frame* (Chicago: University of Chicago Press, 2012), 19–21.

9 “Report of Council, 1947,” in *First Annual Report of the Severn Wildfowl Trust* (Slimbridge, Gloucestershire, 1947), 3.

10 Peter Scott, “To Save the Hawaiian Goose: An Experiment in England,” *The Times*, June 2, 1952; Peter Scott to Paul Breese, August 24, 1961, Papers and Correspondence of Sir Peter Markham Scott (Scott Papers), C.916, Cambridge University Library Department of Archives and Manuscripts, Cambridge, United Kingdom.

11 J. Donald Smith, “The Hawaiian Goose (Nene) Restoration Program,” *Journal of Wildlife Management* 16 (1952): 2.

12 Cristina Bacchilega, *Legendary Hawai‘i and the Politics of Place: Tradition, Translation and Tourism* (Philadelphia: University of Pennsylvania Press, 2007).

13 Andrew C. Isenberg, *The Destruction of the Bison: An Environmental History, 1750–1920* (Cambridge: Cambridge University Press, 2000); Mark V. Barrow Jr., *Nature’s Ghosts: Confronting Extinction from the Age of Jefferson to the Age of Ecology* (Chicago: University of Chicago Press, 2009), 108–34; E. Elena Songster, *Panda Nation: The Construction and Conservation of China’s Modern Icon* (Oxford: Oxford University Press, 2018). On how the meanings we attribute to endangered animals relate to the broader construction of nature, see William J. Cronon, “The Trouble with Wilderness; Or, Getting Back to the Wrong Nature,” in *Uncommon Ground: Rethinking the Human Place in Nature*, ed. William J. Cronon (New York W. W. Norton and Company, 1995), 81–82.

14 On how conservation organizations deploy “ideological mediation” to create or add value to endangered animals, see Elizabeth Garland, “The Elephant in the Room: Confronting the Colonial Character of Wildlife Conservation in Africa,” *African Studies Review* 51 (2008): 63. On how terms such as “native” are ideologically loaded and culturally determined, see Stefan Helmreich, “How Scientists Think About ‘Natives’ for Example: A Problem of Taxonomy among Biologists of Alien Species in Hawaii,” *Journal of the Royal Anthropological Institute* 11 (2005): 107–28.

15 Peter Scott, *The Eye of the Wind* (London: Hodder and Stoughton, 1961), 7.

16 Ibid., 77–78.

17 Peter Scott, “Ne-Nes at the Wildfowl Trust in England,” *Elepaio: Journal of the Hawaiian Audubon Society* 16 (1956): 66–67. There is no archival record of this early correspondence. It is unclear whether Scott knew how endangered the nēnē were or whether he just wanted another rare species for his wildfowl collection.

18 Elspeth Huxley, *Peter Scott: Painter and Naturalist* (London: Faber & Faber, 1993), 158–60.

19 “Report of Council,” 3.

20 Peter Scott, “The Severn Wildfowl Trust: First Booklet,” 1948, reprinted in Paul Walkden, ed., *Peter Scott: Collected Writings, 1933–1989* (Slimbridge: Wildfowl and Wetlands Trust, 2016), 70.

21 Stephen J. Macekura, *Of Limits and Growth: The Rise of Global Sustainable Development in the Twentieth Century* (Cambridge: Cambridge University Press, 2015), 34.

22 Barrow, *Nature’s Ghosts*, 310–25; Martin Holdgate, *The Green Web: A Union for World Conservation* (London: Earthscan, 1999).

23 Hean-Paul Harroy, “Proceedings and Papers” (International Technical Conference on the Protection of Nature, Lake Success, New York, 22–29 August 1949), 135, https://portals.iucn.org/library/node/5938.

24 “Report on Activities of the IUPN 1951–52,” in *Proceedings of the Third General Assembly of the International Union for the Protection of Nature* (Caracas, September 1952), 31; “Fifth Statutory Sitting,” in *Proceedings of the Second General Assembly of the International Union for the Protection of Nature* (Brussels, October 1950), 49. The International Union for the Preservation of Nature was funded by a small stipend from United Nations Educational, Scientific and Cultural Organization and subscriptions from members, which included national governments, conservation organizations, museums, and universities.

25 Harroy, “Proceedings and Papers,” 135.

26 “The Ne-Ne Project,” in *Third Annual Report of the Severn Wildfowl Trust, 1949–1950* (London: Country Life, 1951), 54.

27 Scott, *Eye of the Wind*, 607–8.

28 Charles W. Schwartz and Elizabeth Reeder Schwartz, *A Reconnaissance of the Game Birds of Hawaii* (Territory of Hawaii: Board of Commissioners of Agriculture and Forestry, 1949), 122–23.

29 Smith, “Hawaiian Goose,” 5.

30 Ibid.

31 Ibid., 6.

32 Scott, “Ne-nes at the Wildfowl Trust,” 66–67.

33 Ibid., 66.

34 Peter Scott, “Hawaiian Geese,” in *Sixth Annual Report of the Severn Wildfowl Trust* (London: Country Life, 1953), 32.

35 Thom Van Dooren, *Flight Ways: Life and Loss and the Edge of Extinction* (New York: Columbia University Press, 2014), 88–94.

36 Tommy Johnston to Herbert Shipman, June 10, 1958, Scott Papers, C.916.

37 Van Dooren, “Day with Crows,” 2–5; Vinciane Despret, *What Would Animals Say If We Asked the Right Questions?* (Minneapolis: University of Minnesota Press, 2016), especially 81–88, “K for Killable.”

38 Scott, “To Save the Hawaiian Goose.”

39 Smith, “Hawaiian Goose,” 8.

40 On anthropocentrism and conservation, see Robert Garner, *Animals, Politics and Morality* (Manchester: Manchester University Press, 2003), 162–93.

41 Peter Scott, “Saving the World’s Wildlife,” *BBC Radio*, October 10, 1961, Scott Papers, G6.

42 Peter Scott, “Things of Value,” (talk given to the Institute of Directors Annual Conference, November 5, 1964), Scott Papers, G6 (emphasis in original).

43 Peter Scott, “Species Extinction in Birds” (1972), reprinted in Walkden, *Peter Scott*, 245.

44 Scott, “Things of Value.”

45 Scott, “To Save the Hawaiian Goose.”

46 Smith, “Hawaiian Goose,” 2.

47 Bacchilega, *Legendary Hawai‘i*, 5.

48 Jane Desmond, *Staging Tourism: Bodies on Display from Waikiki to Sea World* (Chicago: University of Chicago Press, 2001), 11, 79–121; Eric Hobsbawm and Terrence Ranger, eds., *The Invention of Tradition* (Cambridge: University of Cambridge Press, 1983, repr. 2013).

49 Sherm Williams, “Hawaiian Nene Not a Gone Goose after All,” *Honolulu Advertiser*, March 29, 1957, Scott Papers, M.330.

50 Paul Breese to Peter Scott, March 22, 1957, Scott Papers, C.915.

51 Resolution Proposed by the Fauna Committee at the Seventh Annual Meeting of the Conservation Council for Hawaii, February 1957, Scott Papers, C.915.

52 Tourist travel grew at an average rate of 20 percent a year during the 1950s. For more detail, see Tom Coffman, *The Island Edge of America: A Political History of Hawai‘i* (Honolulu: University of Hawai‘i Press, 2003), 169–70.

53 This is not to say that statehood was universally endorsed by the 1950s. Some politicians from southern US states opposed the admission of a multiracial territory, while prominent Native Hawaiians resisted statehood and campaigned for full independence. See Dean Itsuji Saranillo, “Colliding Histories: Hawai‘i Statehood at the Intersection of Asians ‘Ineligible to Citizenship’ and Hawaiians ‘Unfit for Self-Government’,” *Journal of Asian American Studies* 13 (2010): 283–309; Roger Bell, *Last among Equals: Hawaiian Statehood and American Politics* (Honolulu: University of Hawai‘i Press, 1984).

54 Resolution Proposed by the Fauna Committee; “Nene Official Bird of Hawaiian Isles,” *Honolulu Star-Bulletin*, May 8, 1957, Scott Papers, C.916.

55 “Extension of Remarks of Hon. John A. Burns, Delegate from Hawaii, in the House of Representatives,” *Congressional Record*—*Appendix*, March 24, 1958, Scott Papers, C.916.

56 Jack Elsen, “U.S. Asked to Save Bird Becoming a Gone Goose,” *Washington Post*, March 25, 1958.

57 Division of Fish and Game, Department of Land and Natural Resources, *Nene Restoration Project Report*, July 1967, Scott Papers, C.920.

58 Paul Breese to Peter Scott, November 13, 1958, Scott Papers, C.917.

59 Valerio Valeri, *Kingship and Sacrifice: Ritual and Society in Ancient Hawaii*, translated by Patricia Wissing (Chicago: University of Chicago Press, 1985), 28.

60 Martha Warren Beckwith, ed. and trans., *Kumulipo: A Hawaiian Creation Chant* (Chicago: University of Chicago Press, 1951), lines 0348–49.

61 Peter Scott to Paul Breese, May 29, 1959, Scott Papers, C.918.

62 Peter Scott to John A. Burns, April 22, 1958, Scott Papers, C.916.

63 Peter Scott to Paul Breese, undated letter, Scott Papers, C.915.

64 Walker, “Nene Restoration Project,” 3; Kear and Berger, *Hawaiian Goose*, 102–3.

65 Lancing Parker to Peter Scott, September 9, 1958, Scott Papers, C.917. While predators threatened the nēnē in this new sanctuary, like the Pohakuloa breeding facility, it was situated above the three hundred-meter limit at which mosquitos that carried the devastating avian pox virus failed to survive. This ensured the restoration project did not face one of the major obstacles that impeded efforts to save birds that lived at lower altitudes, such as the Hawaiian honeycreeper. See Kear and Berger, *Hawaiian Goose*, 86, 119–20.

66 Gerald Durrell to Peter Scott, June 23, 1987, Scott Papers, C.640. On the development of invasion biology, see Daniel Simberloff, “The Rise of Modern Invasion Biology and American Attitudes towards Introduced Species,” in *Invasive and Introduced Plants and Animals: Human Perception, Attitudes and Management*, ed. Ian D. Rotherham and Robert A. Lambert (London: Earthscan, 2011), 127–37.

67 Peter Scott to Paul Breese, June 1, 1958, Scott Papers, C.917. On the long-standing preference for conservation in “the wild,” see Irus Braverman, *Wild Life: The Institution of Nature* (Stanford: Stanford University Press, 2015).

68 Paul Breese to Peter Scott, June 16, 1958, Scott Papers, C.917.

69 Memo from Geoffrey Matthews to Peter Scott, June 1958, Scott Papers, C.917.

70 Peter Scott to Paul Breese, July 31, 1958, Scott Papers, C.917.

71 Peter Scott to Herbert Shipman, May 29, 1959, Scott Papers, C.918; Peter Scott to Paul Breese (draft letter), 1959, Scott Papers, C.917.

72 “Nene Immigrants Arrive,” newspaper clipping, undated, Scott Papers, C.920; Ed Sakamoto, “Travelling, Nene Style, Is Strictly for the Birds,” *Honolulu Advertiser*, August 7, 1965, Scott Papers, C.920.

73 Jerome J. Pratt, “Maui’s Nature Trails,” *Maui News*, September 20, 1969, Scott Papers, C.921.

74 Cobey Black, “The Nene’s Savior: Peter Scott,” *Honolulu Advertiser*, December 4, 1973, Scott Papers, C.921.

75 Ginny Trumbull, “The Return of Hawaii’s Wild Goose,” *National Parks and Conservation Magazine* 50 (1976): 19.

76 J. P. Linduska, assistant director of the US Fish and Wildlife Service, to Peter Scott, July 16, 1973, Scott Papers, C.922.

77 Trumbull, “Return of Hawaii’s Wild Goose,” 20.

78 Peter Scott to Lancing Parker, October 14, 1961, Scott Papers, C.917; Peter Scott to Charles Schwartz, May 29, 1981, Scott Papers, M.2333.

79 Matthew Chrulew, “Managing Love and Death at the Zoo: The Biopolitics of Endangered Species Protection,” *Australian Humanities Review* 50 (2011): 141.

80 Harriet Ritvo, “Species,” in *Critical Terms for Animal Studies*, ed. Lori Gruen (Chicago: University of Chicago Press, 2018), 385.

81 Scott, *Eye of the Wind*, 608.

82 Janet Kear, “The Problems of Breeding Endangered Species in Captivity,” *International Zoo Yearbook* 17 (1977): 7.

83 Kear and Berger, *Hawaiian Goose*, 77, 117.

84 Jamie Lorimer, *Wildlife in the Anthropocene: Conservation after Nature* (Minneapolis: University of Minneapolis Press, 2015), 13. For other works that explore the biopolitical nature of conservation and our treatment of animals more generally, see Braverman, *Wild Life*, 227–32; Wolfe, *Before the Law*.

85 Timothy Cooper and Anna Green, “The *Torrey Canyon* Disaster, Everyday Life and the ‘Greening’ of Britain,” *Environmental History* 22 (2017): 101–26; Alex Loftus, *Everyday Environmentalism: Creating an Urban Political Ecology* (Minneapolis: University of Minnesota Press, 2012).

86 Julia Adeney Thomas, “History and Biology in the Anthropocene: Problems of Scale, Problems of Value,” *American Historical Review* 119 (2014): 1588.

87 Van Dooren, *Flight Ways*, 10–13.

## Notes

***Duncan Wilson*** *is a lecturer at the University of Manchester’s Centre for the History of Science, Technology and Medicine. He has previously worked on the history of bioethics and the history of public attitudes to biological science in the United Kingdom. He is currently writing a book that details the various ways in which conservation organizations valued endangered animals during the twentieth century.*

